# Elicitation of expert prior opinion to design the BARJDM trial in juvenile dermatomyositis

**DOI:** 10.1093/rheumatology/keae392

**Published:** 2024-07-29

**Authors:** Charalampia Papadopoulou, Neil Martin, Nadia Rafiq, Liza McCann, Giulia Varner, Kerstin Nott, Sandrine Compeyrot-Lacassagne, Maria Leandro, Charlene Foley, Kishore Warrier, Nathan Green, Mandy Wan, Hakim-Moulay Dehbi, John Whitehead, Despina Eleftheriou, Paul Brogan

**Affiliations:** Paediatric Rheumatology Department, Great Ormond Street Hospital for Children, NHS Foundation Trust, London, UK; Inflammation and Rheumatology Section, University College London Great Ormond Street Institute of Child Health, London, UK; Paediatric Rheumatology Department, The Royal Hospital for Children, Glasgow, UK; Paediatric Rheumatology Department, The Evelina London Children’s Hospital, London, UK; Paediatric Rheumatology Department, Alder Hey Children’s Hospital, Liverpool, UK; Paediatric Rheumatology Department, Royal Manchester Children’s Hospital, Manchester, UK; Paediatric Rheumatology Department, Southampton Children’s Hospital, Southampton, UK; Paediatric Rheumatology Department, Great Ormond Street Hospital for Children, NHS Foundation Trust, London, UK; Inflammation and Rheumatology Section, University College London Great Ormond Street Institute of Child Health, London, UK; Rheumatology Department, University College London Hospital, London, UK; Centre for Rheumatology, University College London, London, UK; Paediatric Rheumatology Department, The Evelina London Children’s Hospital, London, UK; Paediatric Rheumatology Department, Nottingham Children’s Hospital, Nottingham, UK; Department of Statistical Science, UCL, London, UK; Paediatric Rheumatology Department, The Evelina London Children’s Hospital, London, UK; Institute of Pharmaceutical Science, King's College London, London, UK; Comprehensive Clinical Trials Unit, Institute of Clinical Trials and Methodology, UCL, London, UK; Department of Mathematics and Statistics, Lancaster University, Lancaster, UK; Paediatric Rheumatology Department, Great Ormond Street Hospital for Children, NHS Foundation Trust, London, UK; Inflammation and Rheumatology Section, University College London Great Ormond Street Institute of Child Health, London, UK; Paediatric Rheumatology Department, Great Ormond Street Hospital for Children, NHS Foundation Trust, London, UK; Inflammation and Rheumatology Section, University College London Great Ormond Street Institute of Child Health, London, UK

**Keywords:** JDM, Bayesian trial, prior expert opinion, MTX, baricitinib, BARJDM

## Abstract

**Objectives:**

To elicit and quantify expert opinion concerning the relative merits of two treatments for a rare inflammatory disease: JDM. The formal expression of expert opinion reported in this article will be used in a Bayesian analysis of a forthcoming randomized controlled trial known as BARJDM (baricitinib for JDM).

**Methods:**

A Bayesian prior elicitation meeting was convened, following a previously described methodological template. Opinion was sought on the probability that a patient in the BARJDM trial would achieve clinically inactive disease, off glucocorticoids (GC) within a 12-month period with either MTX (standard of care); or baricitinib (a Janus kinase inhibitor, JAKi), with GC schedules identical in both arms of the trial. Experts’ views were discussed and refined following presentation and further discussion of summated published data regarding efficacy of MTX or JAKi for JDM.

**Results:**

Ten UK paediatric rheumatology consultants (including one adolescent paediatric rheumatologist) participated in the elicitation meeting. All had expertise in JDM, leading active National Health Service clinics for this disease. Consensus expert prior opinion was that the most likely probability of clinically inactive disease off GC within 12 months was 0.55 on baricitinib and 0.23 on MTX, with a greater degree of uncertainty for baricitinib.

**Conclusion:**

Experts currently think that baricitinib is superior to MTX for the treatment of JDM, although there is uncertainty around this. BARJDM will therefore integrate randomized trial data with this expert prior opinion to derive a posterior distribution for the relative efficacy of baricitinib compared with MTX.

Rheumatology key messages

- Experts currently believe that baricitinib is more efficacious than MTX for JDM.
- Prior opinion can be integrated with randomized data to estimate treatment efficacy in Bayesian trials.
- Such Bayesian clinical trials can maximize learning from small clinical trials in rare diseases.

## Introduction

Childhood-onset or juvenile idiopathic inflammatory myopathies (JIIMs) encompass a number of rare yet severe conditions affecting children and young individuals, primarily impacting muscles and skin but potentially involving various organs such as lungs, gut, joints, heart and the central nervous system [1]. The reported incidence ranges between 1.6 and 4.0 cases per million children per year [2]; the estimated prevalence is 2.5 cases per 100 000 children [2], mainly comprised of JDM, seen in over 85% of JIIM cases [3, 4]. Although JDM mortality has decreased globally and is often below 4% [5–7], certain cohorts still exhibit higher mortality rates of 5–8% [8–10]. Over the years, the focus has shifted towards assessing long-term functional outcomes, morbidity and health-related quality of life. The risk of disease damage increases linearly with time since diagnosis, emphasizing the need for early disease control with targeted treatment [11]. Up to 41% of patients experience functional impairment, leading to increased pain and decreased quality of life [6, 7, 9, 10, 12–14]. Children may face growth impairment or delayed puberty, particularly if the active phase of the disease occurs during early puberty. In adulthood, individuals with a history of JDM may still have active myositis requiring continuing immunosuppressive medication [15]. Additionally, JDM is linked to long-term risk of cardiovascular, pulmonary or cerebrovascular diseases [16, 17].

The rarity of JDM has resulted in a lack of randomized controlled clinical trials (RCTs). An international multicentre randomized trial established MTX combined with glucocorticoids (GC) as standard of care [18]. This trial took many years to conduct and needed 54 centres in 22 countries to recruit 139 newly diagnosed JDM patients. Randomization was to one of three different treatments: prednisone alone; prednisone in combination with ciclosporin or prednisone plus MTX. The results were most favourable for the MTX group, which is now accepted as standard of care [18–20]. However, only 26% of patients achieved clinically inactive disease after 12 months of treatment with GC and MTX [18]. Furthermore, responders experienced a lengthy median time to clinical remission (41.9 months), and concerns about GC toxicity persisted since MTX had only a limited steroid sparing effect.

There is, therefore, a clear need for better treatments for JDM. However, high-level evidence regarding the efficacy and safety of new medications primarily derived from case reports or case series [21]. Several clinical trials are currently underway to assess the safety and effectiveness of JAK inhibitors (JAKi) in adults with treatment-resistant myositis [22, 23]. For paediatric patients, there are only retrospective and uncontrolled reports of JAKi use for JDM. These suggest good clinical efficacy and safety and also provide indications that JAKi may target JDM at an immunopathogenic level, with downregulation of IFN biomarkers: normalization of whole blood IFN1 gene signature, and reduction of STAT1 phosphorylation in T cells and monocytes down to healthy control levels [24–26]. These initial observations suggest that JAKi could be an effective and better-targeted treatment for JDM, but no RCT data are available to date.

An RCT leading to a definitive conclusion is infeasible in the UK since too few JDM patients are likely to be available to provide adequate power. For example, a 1:1 randomized trial with power 0.90 to detect an efficacy odds ratio of 2.0 (a reasonable superiority effect size) using a two-sided significance level of 0.05 would require 416 patients (208 per treatment arm). This number would take several decades to recruit in the UK and would be extremely challenging even for an international study.

To provide some quantification of the relative efficacy of MTX (standard of care) vs. the JAKi baricitinib, the consensus opinion of their relative merits was elicited from 10 expert clinicians. This is to be used in the Bayesian analysis of a forthcoming prospective randomized comparison of the two treatments (BARJDM: baricitinib for JDM). This article gives an account of how the expert prior opinion we derived for BARJDM was elicited, using our previously described methodological template [27].

## Materials and methods

### BARJDM trial synopsis

BARJDM is a multicentre, open label, randomized, controlled, superiority trial to assess the effectiveness and safety of baricitinib in combination with GC for the treatment of newly diagnosed JDM, and is currently in set up. The trial will employ Bayesian methodology for the primary end point analysis and will combine the evidence obtained from the patients treated with prior opinion elicited from 10 UK experts. BARJDM will enrol 30 newly diagnosed JDM patients across 5–6 UK centres. Patients will be treated for 12 months, with 10 patients receiving MTX (standard treatment) and 20 patients receiving baricitinib. Patients in both treatment arms will receive the same protocoled GC regimen, weaning off by 6 months. Patient progress will be assessed at 3 and 6 months, with the option of increasing the baricitinib dose at these time points if there is an inadequate improvement according to protocoled definitions. Non-mandated rescue treatments can be used if clinically required, following local standards of care. The primary end point of the trial will be the percentage of patients with inactive disease, off GC, at 12 months (Table 1).

**Table 1.** The baricitinib for JDM (BARJDM) trial design

Hypothesis	Oral baricitinib combined with glucocorticoids is superior to subcutaneous MTX combined with glucocorticoids for the treatment of newly diagnosed JDM
Entry criteria	Newly diagnosed, treatment naïve^a^ JDM patients (≤17 years of age) who meet EULAR/ACR classification criteria for dermatomyositis
Randomization and sample size	2:1 baricitinib (*n* = 20): MTX (*n* = 10); 30 patients total
Control (MTX) treatment arm	MTX dose: 15 mg/m^2^ subcutaneously once a week
Experimental (baricitinib) treatment arm	Baricitinib starting dose, all patients: ≥6 years: 4 mg once a day; <6 years: 2 mg once a day. Dose increases^c^ permitted at 3 or 6 months if lack of clinical response (defined according to protocol)
Glucocorticoid regimen	Same for both arms of the trial: prednisolone 2 mg/kg/day, weaning off completely by 6 months according to a protocol Up to three doses of high dose intravenous methylprednisolone (30 mg/kg/dose) are permitted after randomization
Primary end point	Percentage achieving clinically inactive disease^b^, and off glucocorticoids at 12 months
Rescue treatment (non-mandatory)	At any stage of the study, all patients who develop clinical deterioration can receive rescue treatment as per treating physician decision Receipt of rescue treatment will be deemed as treatment failure in terms of the primary end point analysis

a Treatment naïve: patients who received prednisolone no higher than 1 mg/kg/day for up to 4 weeks can be included; patients who received IV methylprednisolone up to 30 mg/kg/day for 3 days prior to enrolment can be included; patients who have received MTX or baricitinib prior to enrolment will be excluded.

b Clinically inactive disease is defined using modified PRINTO criteria: no active skin disease; creatine kinase (CK) ≤150 U/l; childhood myositis assessment scale (CMAS) ≥48/52; Manual Muscle Testing 8 (MMT8) ≥78/80; physician global assessment (PhyGLOVAS) ≤0.2/10 on a visual analogue scale.

c Dose increased to that described for monogenetic interferonopathy (https://www.england.nhs.uk/wp-content/uploads/2021/07/1930-Baricitinib-policy-Final-1.pdf).

### Identifying and inviting clinical experts

A face-to-face meeting of UK JDM experts was convened on 29–30 August 2023 at UCL, London, UK. An expert was defined as a paediatric or adolescent rheumatology consultant practicing in the UK, with an interest in and experience of looking after children and young people with JDM evidenced by having seen on average of at least one new case per year throughout their practise; and active participation or leadership of a National Health Service clinical service managing patients with JDM. Invitations were sent to their professional email addresses found through the Juvenile Cohort and Biomarker Study (JDCBS) [5] and through the national JDM topic specific group (TSG) of the Clinical Studies Group. Initial expressions of interest were received from 16 eligible respondents, of whom (for logistical reasons) 10 were able to attend the face-to-face meeting. The expert group comprised the following co-authors of this article: N.M., N.R., L.M., G.V., K.N., S.L., C.F., M.L., C.P., K.W., C.P., K.W. They came from across the UK (see author affiliations). All participants volunteered to take part in the prior elicitation meeting as experts. No patients were involved in the meeting. Ethics approval was therefore not required.

### Selection of the specific quantities to be elicited

Expert opinion was sought on the probability that a patient satisfying the entry criteria for BARJDM (newly diagnosed, treatment naïve) would succeed according to the primary end point: clinically inactive disease, off GC at 12 months. The probability of success for a patient treated with MTX was denoted by *p_C_*, and expert views on the value of *p_C_* were elicited directly. The corresponding probability of successful treatment with baricitinib was denoted by *p_E_*, the value of which was derived indirectly from expert clinicians using questions about the relative merits of the two drugs, as previously described [27, 28]. Note that the application in these earlier articles was to a study seeking to demonstrate the non-inferiority of a study drug, whereas here the objective is to find evidence of superiority. This means that the mathematical details here differed slightly from those reported earlier, being easier to work through.

### Mathematical modelling of beliefs and uncertainties

**Figure 1.**
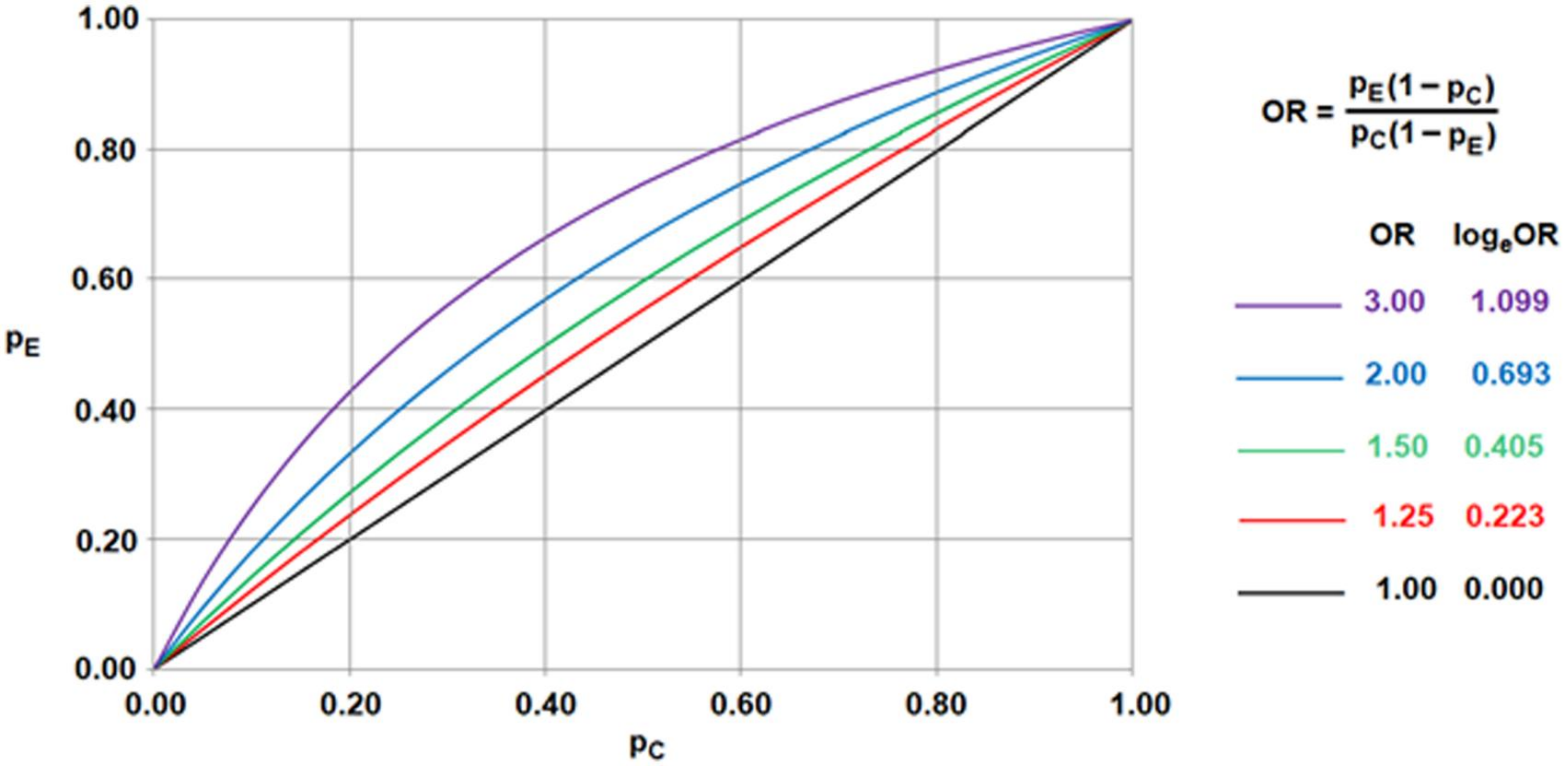
Definition and interpretation of odds ratios. For example, if the odds ratio is 2.00 and *p_C_* equals 0.40, then *p_E_* will be equal to 0.57. Notice that, for every odds ratio and every value of *p_C_*, there is a value of *p_E_* between 0 and 1

Expert opinions were elicited using the questionnaire shown in Table 2. Opinions regarding the value of *p_C_* were determined by two parameters aimed at capturing the experts' preferences for the most probable *p_C_* value and their associated uncertainty levels. These values were sought by asking the experts two questions (Q1 and Q2). The relative efficacy of MTX and baricitinib was expressed in terms of the odds ratio (OR) and the log-odds ratio (*θ* = log_e_OR, the log being to base e, otherwise known as the natural log). The definition of OR and its implications for comparisons of *p_E_* and *p_C_* are shown in Fig. 1. The value of *θ* was modelled as a normal distribution, the parameters of which were determined by asking experts two questions (Q3 and Q4). From the resulting opinions about *p_C_* and *θ*, a consistent opinion about *p_Ε_* was derived. These two parameters were used as they could be treated as independent: experts’ opinions about *p_C_* and *p_E_* would not be independent of one another and thus more difficult to elicit. To ensure that the elicited opinion truly reflected the experts' personal beliefs, two additional reworded ‘sense-check’ questions were posed (Q5 and Q6) and used informally in discussions. The calculation of the effective sample size (ESS) of a prior distribution proved particularly beneficial for clinicians in assessing their prior beliefs. This metric essentially provided clinicians with the hypothetical number of patients in a clinical trial needed to statistically yield the prior level of certainty being expressed for the parameters. Further details on these calculations and comparisons with alternative approaches can be found in [28].

**Table 2.** Establishing the BARJDM expert prior opinion

*Response rate on MTX*
Q1: I think the response rate on MTX will be 0______
Q2: I am 75% sure the response rate on MTX will be <0______

***Improvement due to baricitinib***

Q3: I think that the odds ratio on response for baricitinib relative to MTX will exceed 1.0 with probability ______%
Q4: I think that the odds ratio on response for baricitinib relative to MTX will exceed 2.0 with probability ______%

***Response rate on baricitinib (reworded ‘sense-check’ questions)***

Q5: I think the response rate on baricitinib will be 0______
Q6: I am 75% sure the response rate on baricitinib will be <0______

Definition of response: For the BARJDM trial a patient will be deemed to have responded if their disease is clinically inactive and they are off glucocorticoids at the assessment made 12 months after randomization to treatment; Q: question.

The elicitation procedure followed an iterative approach, facilitated by mathematical models and customized computer software that enabled the generation of rapid and lucid graphical representations of density functions to facilitate discussion in real time. The presentation also included a demonstration of the outcomes resulting from the incorporation of hypothetical datasets to form posterior distributions. The computer program, developed in R using the Shiny package, was designed to offer a user-friendly and interactive interface, as detailed in earlier descriptions [27]. A testing phase involving two clinicians (P.B. and D.E.), who consequently did not contribute their prior opinions during the actual meeting, led to refinements and adjustments before the formal meeting took place. The final software package we developed is freely available in editable format at: https://egon.stats.ucl.ac.uk/projects/TrialExpertElicitation/.

### Training of the expert participants

**Figure 2.**
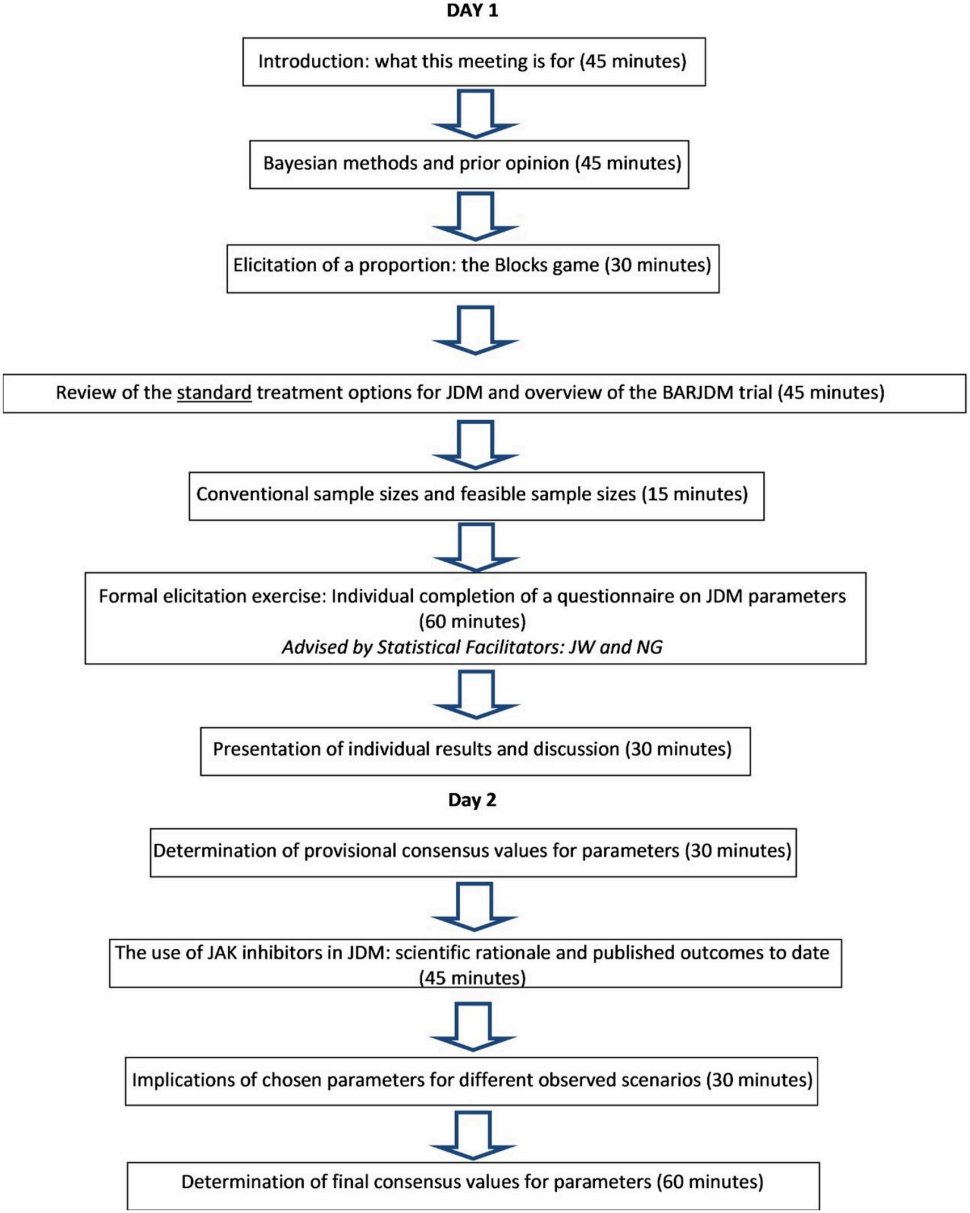
BARJDM (baricitinib for JDM) prior opinion elicitation meeting

Fig. 2 outlines the two-day meeting activities and their respective time allocations. Throughout the session, two statistical facilitators (J.W. and N.G.) supported the elicitation process. The meeting commenced with one of the statistical facilitators (J.W.) delivering a talk introducing Bayesian reasoning, credible intervals and the representation of treatment differences as log-odds ratios. This was followed by a practical exercise involving guessing the proportion of black blocks in a jar containing 60 pink and black blocks. The same exercise is described in [26]. It served as a rehearsal for the elicitation process and Bayesian methods in a neutral setting. Following the practical exercise, a talk was given summarizing the standard treatment options for JDM and providing an overview of the BARJDM trial.

### Elicitation of opinions

Experts individually completed the structured questionnaire (Table 2), without discussion with any of the other experts. Each expert then discussed their answers privately with one of the statistical facilitators. The latter used the R program to visualize answers and guide each expert to adjust their responses until satisfied that the final model represented their opinions.

Consensus prior distributions for *θ* and *p_C_* were then sought in a plenary session of all experts. Rather than using automatic mathematical aggregation or multiple priors, each expert presented their finalized questionnaire responses. Lengthy discussions led to a first attempt at reaching a consensus. This group prior reflected greater uncertainty about the relative efficacy of the two treatment arms than the individual priors, taking account of the range of opinions expressed and being influenced by the ESS which demonstrated how precise prior opinions would be equivalent to information from very large clinical studies. Potential BARJDM study outcomes for 30 patients were considered, and posterior distributions illustrated the relative impact of prior beliefs and data. After overnight reflection, a final provisional consensus was reached at the beginning of the second day.

### Presentation of published outcomes on the use of JAK inhibitors in JDM and influence on the provisional prior opinion

After submitting their individual prior opinion results and agreeing on a provisional consensus opinion, on day 2 the experts were given an update on currently published studies regarding efficacy and safety of JAKi in juvenile IIM (summarized in Supplementary Table S1, available at *Rheumatology* online). This included an overview of efficacy of JAKi used in 176 JDM patients with refractory disease, with specific emphasis on the efficacy across different organ systems (muscles, skin and lungs). Prior distributions from the first day were then updated by the experts to derive a final consensus opinion (defined a priori as ≥80% agreement) regarding the relative efficacy of MTX vs. baricitinib.

Opinion about the randomization allocation ratio that should be used in the trial was also sought; but views about the total sample size were not, since this number was considered fixed (at *n* = 30) by a national feasibility exercise that had been conducted separately based on JDCBS figures informing recruitment projections over 36 months from each of six potentially recruiting sites (allowing for up to 30% recruitment failure rate; data not shown).

## Results

### Provisional expert opinion prior to presentation of published literature

The provisional expert prior opinion derived at the end of day 1 before the presentation of published results regarding the efficacy of JAKi is summarized in Table 3. This suggested a probability of response of 0.23 for MTX, compared with 0.41 for baricitinib, with an ESS of 30 patients (assuming 15 per treatment arm for this purpose).

**Table 3.** Provisional and final consensus prior opinions regarding response rates for MTX and baricitinib

	Provisional consensus opinion	Final consensus opinion (after discussion of literature review)
*Elicited values*		
Response rate on MTX (*p_C_*) (Q1)	0.23	0.23
*p_C_* 75th percentile (Q2)	0.33	0.33
*P* (odds ratio >1.0) (Q3)	87%	90%
*P* (odds ratio >2.0) (Q4)	60%	70%
*Derived values*		
Response rate on baricitinib (*p_E_*)	0.41	0.55
Effective sample size	30	22
Agreement among 10 experts	100%	100%

Provisional consensus opinion (100% agreement) obtained at the end of day 1, prior to the presentation of published literature on the use of Janus kinase inhibitors is shown. On day 2, following presentation and discussion of the published literature (Supplementary Table S1, available at *Rheumatology* online), the final consensus expert prior opinion was derived, again with 100% agreement among the experts. *p_C_* = probability of response to control treatment (MTX), odds ratio = (odds of response to baricitinib)/(odds of response to MTX), *p_E_* = probability of response to experimental treatment (baricitinib).

### Final consensus opinion derived after presentation and discussion of published literature

**Figure 3.**
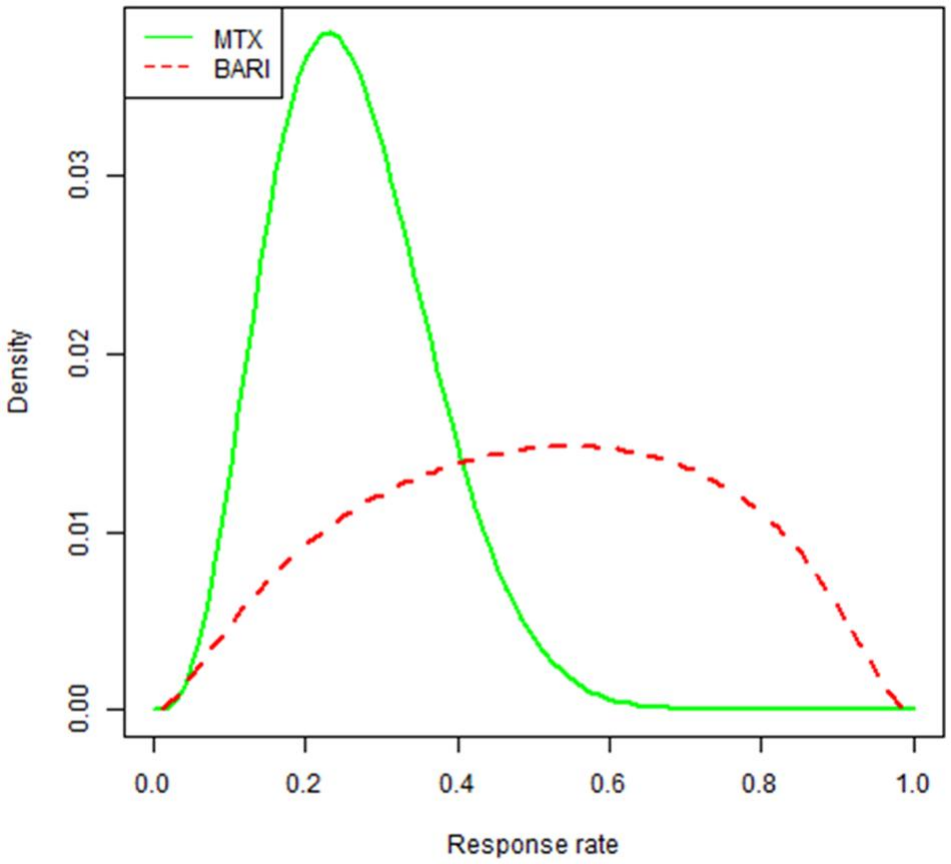
Final consensus expert prior opinion regarding successful response rates (clinically inactive disease at 12 months and off glucocorticoids) for MTX or baricitinib in JDM. The final expert consensus prior opinion was that the most likely value for *p_C_* was 0.23; 90% and 50% credibility intervals were (0.11, 0.45) and (0.19, 0.33), respectively. The prior for *p_E_* is derived from those for *p_C_* and *θ*. It had mode = 0.55; 90% and 50% credibility intervals were (0.18, 0.86) and (0.37, 0.69), respectively and carried the same weight as observing 22 patients, 11 per treatment arm. Bari: baricitinib

The final consensus expert opinion reached at the end of day 2 is also summarized in Table 3 and the corresponding distributions are displayed graphically in Fig. 3. Experts modified their final responses in relation to the efficacy of baricitinib (but not MTX) after extensive discussion of the literature review findings (Supplementary Table S1, available at *Rheumatology* online). In both the provisional and the final judgements, the probability of response to MTX was predicted to be 0.23, with 75% certainty that it will be <0.33. Presentation and open discussion of the literature review (Supplementary Table S1, available at *Rheumatology* online) influenced the final consensus opinion concerning baricitinib. The probability of response on that treatment was finally estimated to be 0.55. There was 90% certainty that baricitinib is better than MTX, and 70% certainty that it is better to the extent that the odds ratio on success exceeds 2.0. This prior opinion carried the same weight (ESS) as observing 22 patients (11 per treatment arm), reflecting an understandable degree of uncertainty among experts regarding the use of baricitinib for JDM given the relatively limited evidence base.

### Randomization allocation

Experts agreed unanimously that even though the consensus prior opinion favoured baricitinib for JDM, there was sufficient equipoise to warrant a randomized controlled trial design for BARJDM. Since much more is known about the use of MTX in JDM compared with baricitinib, a 2:1 baricitinib: MTX randomization allocation was proposed to the experts. This proposal was unanimously accepted.

## Discussion

Conducting clinical trials in rare diseases are challenging due to difficulties recruiting adequate sample size to achieve statistical power. Historically, this has been a major barrier in rare paediatric autoimmune diseases such as JDM. The BARJDM trial will therefore use a Bayesian approach to combine prior expert opinion with the results of our trial to formulate a final result. Such rare disease trial methodology has been used before in paediatric rheumatology [27], and unblocks an important barrier for testing novel JDM-targeted therapeutics. This approach is clearly not as high in the hierarchy of levels of evidence as a trial involving hundreds of patients but does still allow a worthwhile increment in clinically meaningful knowledge to be made. Moreover, the consensus opinion of our 10 experts that are given in this article represent a helpful guide, particularly for less experienced clinicians.

It took some practice for the experts to provide realistic answers to Q3 and Q4 of the questionnaire. Q3 was ‘I think that the odds ratio on response for baricitinib relative to MTX will exceed 1.0 with probability ______%’, and Q4 was the same but with 2.0 in place of 1.0. Once it was appreciated that the difference between the two answers (Q3–Q4) was the belief that the OR lay between 1.0 and 2.0, the questions became easier to address. First, it made it clear that the answer to Q3 had to be larger than that to Q4. Second, it indicated that if the difference was large then the opinion about the value of OR was relatively precise (pretty sure it lies between 1.0 and 2.0), whereas a small difference indicated substantial uncertainty about the relative merits of the two treatments. In the context of the meeting, the answers to Q3 were invariably close to 1. In further applications of this approach, it may be possible to improve the form of Q3 and Q4 to make them easier to answer.

Expert prior opinion exercises of the type reported here come with inherent limitations. Expert opinion is by its nature subjective, and in rare diseases will be expressed with substantial uncertainty. Heterogeneity among experts will lead to a consensus view that has even greater uncertainty than the individual opinions. Experts will face challenges in quantifying their subjective beliefs in a suitable mathematical form, and practice in Bayesian reasoning is therefore a crucial component of any expert prior opinion elicitation exercise [27, 29, 30].

We attempted to mitigate some of these inherent limitations by deriving expert opinion before and after presentation and discussion of the literature regarding treatment of JDM with MTX or JAKi. Unsurprisingly, this did influence the expert prior opinion regarding the use of baricitinib but had no impact on the prior beliefs regarding the efficacy of MTX for JDM (Table 3), since clinicians are much more familiar with the use of MTX for JDM than baricitinib. Although publication bias may influence experts to be overly optimistic regarding efficacy of a new treatment, we did observe a healthy and realistic degree of uncertainty regarding the relative efficacy of baricitinib for JDM, evidenced by the final result associated with an ESS of 22; and the relatively wide distribution regarding the probability of response for baricitinib compared with MTX depicted in Fig. 3.

In conclusion, we applied a previously used approach for derivation of expert prior opinion as part of a Bayesian RCT of baricitinib for JDM. Expert opinion was that baricitinib is likely to be more efficacious than MTX, but with a considerable degree of uncertainty. These prior distributions will be combined with trial data from BARJDM, to derive posterior distributions [31], reported with credible intervals and other numerical and graphical summaries of the relative merits of the two treatments.

## Supplementary Material

keae392_Supplementary_Data

## Data Availability

The data underlying this article are available in the article and in its online supplementary material.
