# Supplementary material for: Elicitation of expert prior opinion to design the BARJDM trial in juvenile dermatomyositis
Source: Rheumatology (Oxford). 2024 Jul 29;63(12):3271–8. doi: 10.1093/rheumatology/keae392 (PMC11637550; doi:10.1093/rheumatology/keae392)
Supplement: keae392_Supplementary_Data [file keae392_supplementary_data.docx]

**SupplementaryTable 1: Proof of concept evidence**

| **JAK Inhibitors in JDM** | | | | | | | |
| --- | --- | --- | --- | --- | --- | --- | --- |
| **No** | **Author** | **Year** | **Cases** | **Disease course** | **Previous treatments** | **JAK inhibitor** | **Outcome** |
| (1) | Sabbagh | 2019 | 2 cases MDA5+ JDM | refractory JDM | CS, MTX, IVIG, abatacept, MMF, rituximab | Tofacitinib | Improvement PGA, MMT8, CDASI, DAS, CHAQ, CT and PFT findings, IFN gene assay, pSTAT1 |
| (2) | Sozeri | 2020 | 2 case refractory JDM | refractory JDM | CS, MTX, cyclophosphamide, pamindronate, MMF, infliximab, | Tofacitinib | After 3 months of therapy, the first patient achieved a complete response (all of the calcifications improved, CK normalized and CMAS score was found to be 52) and the second one achieved moderate response (>50% improvement in calcifications in skin examination and X-ray, CK normalized and CMAS score was found to be 50). |
| (3) | Yu | 2021 | 3 cases refractory JDM | refractory JDM | CS, HCQ, MTX, CSA, MMF, | Tofacitinib | Improvement PGA, MMT8, CMAS, DAS, CHAQ, |
| (4) | Papadopoulou | 2019 | 1 case refractory JDM | refractory JDM | CS, HCQ, MTX, CSA, MMF, CYCLO, abatacept, rituximab, infliximab | Baricitinib | Improvement PGA, MMT8, CMAS, CHAQm cytokines, IFN gene assay, CEC, pSTAT1 |
| (5) | Kim | 2020 | 4 cases refractory JDM | refractory JDM JAGA trial | 3 to 6 immunomodulatory medications | Baricitinib | PGA, MMT8, Patient/Parent GloVAS+H38, and Extramuscular Global Activity, and CDASI, ACR/EULAR Myositis Response Criteria, IFN gene assay, pSTAT1 |
| (6) | Aeschlimann | 2018 | 1 case refractory JDM | refractory JDM | CS, IVIG, PE, MTX, MMF, rituximab, | Ruxolitinib | MMT8, CMAS, PHYGloVAS, Ifn related gene assay, IFNa, pSTAT1 |
| (7) | Le Voyer | 2021 | 10 cases refractory JDM | refractory JDM | CS, 3-6 DMARDS, IVIG, PE | 7 ruxolitinib, 3 baricitinib | clinically inactive disease as per PRINTO, skin DAS, IFNa, Partial response was defined as an improvement of the muscle and/or skin score assessed by MMT/CMAS and skin DAS, respectively, allowing a significant tapering of steroids dosage of at least 50% of the initial dosage |
| (8) | Ding | 2020 | 25 cases refractory JDM | refractory JDM | several immunosuppressive treatents | 7 tofacitinib, 18 ruxolitinib | Cutaneous Assessment Tool Binary Method score |
| (9) | Heinen | 2021 | 1 case refractory JDM | refractory JDM | CS, HCQ, MTX, IVIG, CYCLO, Rituximab | Ruxolitinib | IFN gene assay, CK |
| (10) | Huang | 2023 | 101 cases refractory JDM | Refractory JDM | CS, IVIG, DMARDS, biologics | 77 tofacitinib, 23 ruxolitinib | 65.5% of the patients had improved rashes, and disease activity scores decreased. Overall, 39.6% of JDM patients eliminated glucocorticoids. Muscle strength was improved in all patients who had abnormal muscle strength before JAKi use. Patients and parents provided positive subjective reviews of JAKi, and no serious adverse events were reported. |

Abbreviations: ACR; American college of rheumatology, CDASI; cutaneous dermatomyositis disease area and severity index, CEC; circulating endothelial cells, CHAQ; childhood health assessment questionnaire, CK; creatine kinase, CMAS; childhood myositis assessment score, CS; corticosteroids, CSA; ciclosporin, CT; computed tomography, CYCLO; cyclophosphamide, DAS; disease activity score, DMARDS; disease-modifying antirheumatic drugs, EULAR; European Alliance of associations for rheumatology, GloVAS; global visual analogue score, HCQ; hydroxychloroquine, IFN; interferon, IVIG; intravenous immunoglobulin, JAKi; Januse Kinase inhibitor, JDM; juvenile dermatomyositis, MDA5; melanoma differantiation-associated gene 5, MMF; mycophenolate mofetil, MMT8; manual muscle test 8, MTX; methotrexate, PE; plasma exchange, PFT; pulmonary function test, PGA; physician global assessment, PHYGloVAS; physicial global visual analogue score, PRINTO; Paediatric Rheumatology International Trials Organisation, pSTAT1; phosphorylated STAT1

References

1. Sabbagh S, Almeida de Jesus A, Hwang S, Kuehn HS, Kim H, Jung L, et al. Treatment of anti-MDA5 autoantibody-positive juvenile dermatomyositis using tofacitinib. Brain. 2019;142(11):e59.

2. Sozeri B, Demir F. A striking treatment option for recalcitrant calcinosis in juvenile dermatomyositis: tofacitinib citrate. Rheumatology (Oxford). 2020;59(12):e140-e1.

3. Yu Z, Wang L, Quan M, Zhang T, Song H. Successful management with Janus kinase inhibitor tofacitinib in refractory juvenile dermatomyositis: a pilot study and literature review. Rheumatology (Oxford). 2021;60(4):1700-7.

4. Papadopoulou C, Hong Y, Omoyinmi E, Brogan PA, Eleftheriou D. Janus kinase 1/2 inhibition with baricitinib in the treatment of juvenile dermatomyositis. Brain. 2019;142(3):e8.

5. Kim H, Dill S, O'Brien M, Vian L, Li X, Manukyan M, et al. Janus kinase (JAK) inhibition with baricitinib in refractory juvenile dermatomyositis. Ann Rheum Dis. 2021;80(3):406-8.

6. Aeschlimann FA, Fremond ML, Duffy D, Rice GI, Charuel JL, Bondet V, et al. A child with severe juvenile dermatomyositis treated with ruxolitinib. Brain. 2018;141(11):e80.

7. Le Voyer T, Gitiaux C, Authier FJ, Bodemer C, Melki I, Quartier P, et al. JAK inhibitors are effective in a subset of patients with juvenile dermatomyositis: a monocentric retrospective study. Rheumatology (Oxford). 2021;60(12):5801-8.

8. Ding Y, Huang B, Wang Y, Hou J, Chi Y, Zhou Z, et al. Janus kinase inhibitor significantly improved rash and muscle strength in juvenile dermatomyositis. Ann Rheum Dis. 2021;80(4):543-5.

9. Heinen A, Schnabel A, Bruck N, Smitka M, Wolf C, Lucas N, et al. Interferon signature guiding therapeutic decision making: ruxolitinib as first-line therapy for severe juvenile dermatomyositis? Rheumatology (Oxford). 2021;60(4):e136-e8.

10. Huang B, Wang X, Niu Y, Ding Y, Wang X, Tan Q, et al. Long-term follow-up of Janus-kinase inhibitor and novel active disease biomarker in juvenile dermatomyositis. Rheumatology (Oxford). 2023;62(3):1227-37.
